# Pfam: The protein families database in 2021

**DOI:** 10.1093/nar/gkaa913

**Published:** 2020-10-30

**Authors:** Jaina Mistry, Sara Chuguransky, Lowri Williams, Matloob Qureshi, Gustavo A Salazar, Erik L L Sonnhammer, Silvio C E Tosatto, Lisanna Paladin, Shriya Raj, Lorna J Richardson, Robert D Finn, Alex Bateman

**Affiliations:** European Molecular Biology Laboratory, European Bioinformatics Institute (EMBL-EBI), Wellcome Genome Campus, Hinxton CB10 1SD, UK; European Molecular Biology Laboratory, European Bioinformatics Institute (EMBL-EBI), Wellcome Genome Campus, Hinxton CB10 1SD, UK; European Molecular Biology Laboratory, European Bioinformatics Institute (EMBL-EBI), Wellcome Genome Campus, Hinxton CB10 1SD, UK; European Molecular Biology Laboratory, European Bioinformatics Institute (EMBL-EBI), Wellcome Genome Campus, Hinxton CB10 1SD, UK; European Molecular Biology Laboratory, European Bioinformatics Institute (EMBL-EBI), Wellcome Genome Campus, Hinxton CB10 1SD, UK; Department of Biochemistry and Biophysics, Science for Life Laboratory, Stockholm University, Box 1031, 17121 Solna, Sweden; Department of Biomedical Sciences, University of Padua, 35131 Padova, Italy; Department of Biomedical Sciences, University of Padua, 35131 Padova, Italy; European Molecular Biology Laboratory, European Bioinformatics Institute (EMBL-EBI), Wellcome Genome Campus, Hinxton CB10 1SD, UK; European Molecular Biology Laboratory, European Bioinformatics Institute (EMBL-EBI), Wellcome Genome Campus, Hinxton CB10 1SD, UK; European Molecular Biology Laboratory, European Bioinformatics Institute (EMBL-EBI), Wellcome Genome Campus, Hinxton CB10 1SD, UK; European Molecular Biology Laboratory, European Bioinformatics Institute (EMBL-EBI), Wellcome Genome Campus, Hinxton CB10 1SD, UK

## Abstract

The Pfam database is a widely used resource for classifying protein sequences into families and domains. Since Pfam was last described in this journal, over 350 new families have been added in Pfam 33.1 and numerous improvements have been made to existing entries. To facilitate research on COVID-19, we have revised the Pfam entries that cover the SARS-CoV-2 proteome, and built new entries for regions that were not covered by Pfam. We have reintroduced Pfam-B which provides an automatically generated supplement to Pfam and contains 136 730 novel clusters of sequences that are not yet matched by a Pfam family. The new Pfam-B is based on a clustering by the MMseqs2 software. We have compared all of the regions in the RepeatsDB to those in Pfam and have started to use the results to build and refine Pfam repeat families. Pfam is freely available for browsing and download at http://pfam.xfam.org/.

## INTRODUCTION

Pfam is a database of protein families and domains that is widely used to analyse novel genomes, metagenomes and to guide experimental work on particular proteins and systems (1,2). Each Pfam family has a seed alignment that contains a representative set of sequences for the entry. A profile hidden Markov model (HMM) is automatically built from the seed alignment and searched against a sequence database called *pfamseq* using the HMMER software (http://hmmer.org/). All sequence regions that satisfy a family-specific curated threshold, also known as the gathering threshold, are aligned to the profile HMM to create the full alignment. It is worth noting that a common misuse of Pfam is to use a single *E*-value threshold across all Pfam HMMs, which results in lower sensitivity and an increase in false positive matches when compared to using the per-family gathering thresholds. Pfam entries are manually annotated with functional information from the literature where available.

Since Pfam release 29.0, *pfamseq* is based on UniProtKB reference proteomes, whilst prior to that, it was based on the whole of UniProtKB (3,4). Although the underlying sequence database is based on reference proteomes, all of the profile HMMs are also searched against UniProtKB and the resulting matches are made available on the Pfam website and in a flatfile format. Similarly, the match data for representative proteomes (5) are provided in the same way. Pfam can be accessed via the website at https://pfam.xfam.org. The flatfiles and exports of the Pfam MySQL database are provided under a CC0 license for each release of the database, and can be found on the FTP site ftp://ftp.ebi.ac.uk/pub/databases/Pfam/releases.

When a Pfam entry is built, we typically search it iteratively against *pfamseq* in order to find more distant homologues. Pfam entries are built such that there are no overlaps between them; this means that the same region of a sequence should not match more than one family. This non-overlap rule proves to be an excellent quality control criteria which helps to avoid including false positive matches into a family. From Pfam 28.0, we relaxed this rule to allow small overlaps between families as it had become increasingly time-consuming to resolve all such overlaps each time we updated *pfamseq* (see (3) for more details).

Sets of Pfam entries that we believe to be evolutionarily related are grouped together into clans (6). Relationships between entries are identified through sequence similarity, structural similarity, functional similarity and/or profile-profile comparisons using software such as HHsearch (7). Where possible, we build a single comprehensive profile HMM to detect all members of a family. For some of the larger superfamilies where this is not possible, we build multiple profile HMMs and put them in the same clan. As families in a clan are evolutionarily related, we allow them to overlap with other members of the same clan. The clans are competed such that if there are multiple overlapping profile HMM matches to families from a single clan, only the match with the lowest *E*-value is displayed on the website, or included in the full alignment of the entry.

Here we describe Pfam 33.1 and some of the family updates we have made since the last release. We also detail the Pfam coverage of the SARS-CoV-2 proteome, and the new method to create Pfam-B which we re-introduced in Pfam 33.1. Finally we describe the results of the analysis we carried out on comparing repeats in the Pfam database to RepeatsDB.

## PFAM 33.1 RELEASE

Pfam 33.0 was due to be released in March 2020, however due to the global COVID-19 pandemic, we delayed its release so that we could focus on improving our SARS-CoV-2 models (see ‘COVID-19 updates’ section for more detail). The updated SARS-CoV-2 models, and a handful of other new Pfam entries were added to Pfam 33.0 to create Pfam 33.1, which was released in May 2020. Release 33.0 was never officially released on the Pfam website, although the database files and flat files are made available on the ftp site.

**Figure 1.**
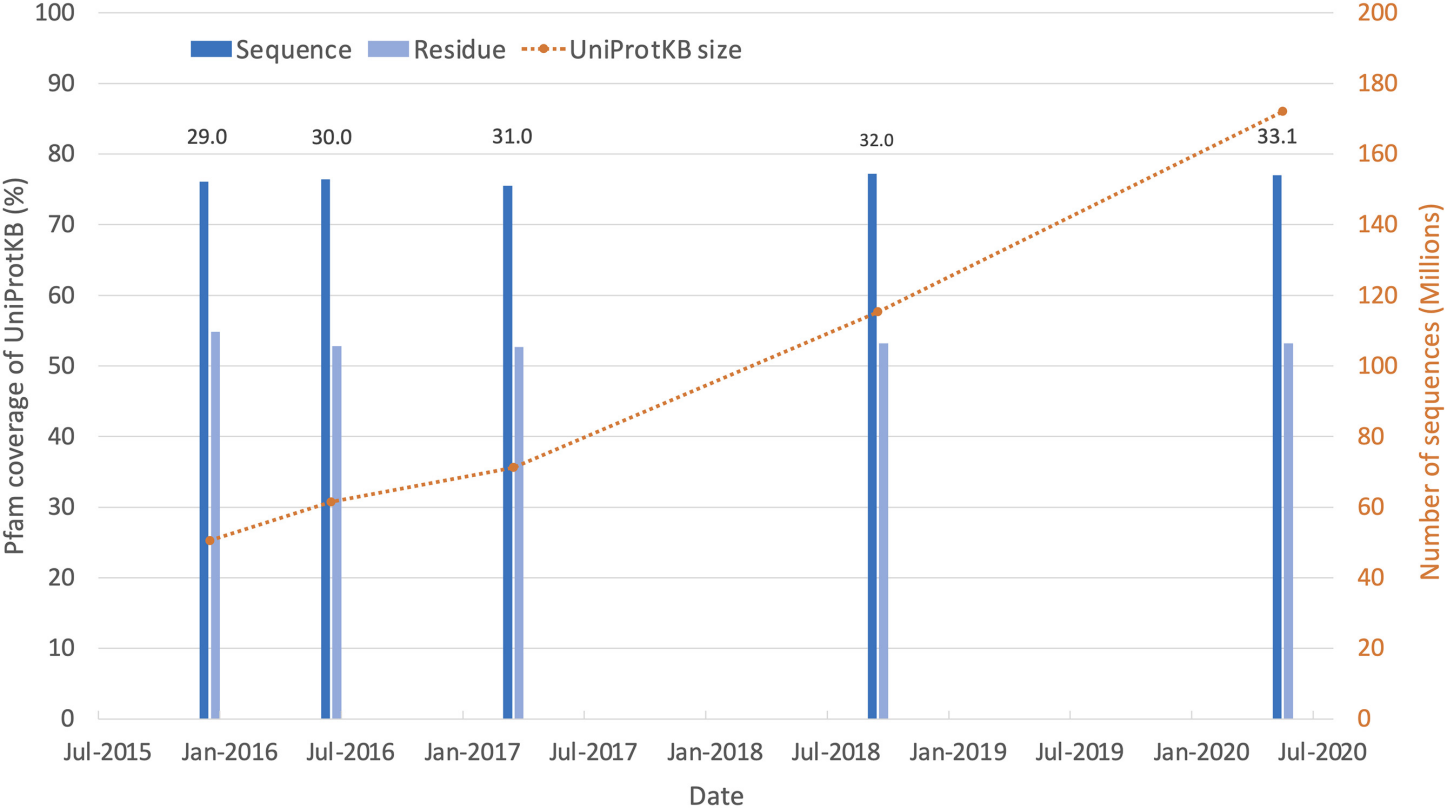
Growth of UniProtKB, and its coverage by Pfam over the last five Pfam releases. As UniProtKB grows in size, the Pfam sequence and residue coverage is maintained at ∼77 and ∼53%, respectively. The UniProtKB size in the figure corresponds to the version of UniProtKB we used for each Pfam release.

Pfam 33.1 contains 18 259 families and 635 clans. Since Pfam 32.0, we have built 355 new families, deleted 25 families and built 8 new clans. Just over 39% of all Pfam families are placed within a clan. Of the sequences in UniProtKB, 77.0% have at least one match to a Pfam entry, and 53.2% residues in UniProtKB fall within a Pfam entry. These figures, termed the sequence and residue coverage respectively, have remained fairly constant over the past five Pfam releases despite a 240% increase in UniProtKB size during that time (see Figure 1). Since 2015, highly redundant bacterial proteomes have been identified and removed from UniProtKB (8). This process ensures a level of diversity in the sequences added to UniProtKB, and prevents, for example, multiple strains of a particular species being added. Pfam is able to maintain a sequence coverage of ∼77%, and a residue coverage of ∼53% largely due to the new sequences being added to UniProtKB matching existing Pfam models. As UniProtKB grows, it is progressively harder to increase the Pfam coverage since newer Pfam entries tend to cover a small taxonomic range. Although new models add little in terms of coverage, they may represent medically important proteins. For example, during our SARS-CoV-2 model improvements, we built the new entry Pfam:PF19213, which corresponds to non-structural protein 6 (NSP6), a protein involved in autophagosome generation.

The Pfam 33.1 sequence and residue coverage of UniProtKB reference proteomes is 75.1 and 49.4%, respectively (slightly lower than the figure for all of UniProtKB mentioned above). This is a 0.6% increase in sequence coverage, and 0.7% decrease in residue coverage compared to Pfam 32.0. Although there was only a 3% increase in the number of sequences in reference proteomes between Pfam releases 32.0 and 33.1, around 2000 bacterial proteomes were removed from the reference proteomes (due to quality issues) during this time. The changes in coverage since Pfam 32.0 are likely to be due to the change in sequences within the reference proteomes set.

## FAMILY IMPROVEMENTS

Despite almost 25 years of active curation, there remain many protein domains and families that are as yet unclassified by Pfam. New families added to Pfam are created from a range of sources, including Pfam-B families and protein structure. Pfam-B alignments in particular have been a very fruitful substrate for families, with historically nearly a third of all Pfam entries being built from them. The new Pfam-B (described below) was used to build 18 entries in Pfam 33.1. We expect that Pfam-B will again become a very useful source of additional families in the coming years.

Protein structures from databases such as the Protein Data Bank (PDB) (9,10) are particularly amenable as a source of new Pfam entries because the Pfam domain boundaries can be defined precisely from the structure. There is often an associated paper to the structure which we use to help annotate the Pfam entry. Building families using protein structures is an ongoing activity, and some of the new SARS-CoV-2 families were built in this way. Additionally, we have made 37 new families based on clusters of sequences from the MGnify metagenomic protein database (11). The MGnify clusters may be another large source from which we could build families from in the future. Using metagenomics clusters can help to cover regions of sequence space that are not well covered by existing genomic sequencing projects.

Pfam has created numerous entries for domains of unknown function (DUF) and uncharacterized protein families (UPF). Over time the functions of some of these are discovered. We continue to scan the literature to identify newly identified functions as well as receiving updates from the community via our helpdesk. Many functions are also identified by the InterPro database (12) update process which checks whether the UniProtKB/Swiss-Prot descriptions of proteins in each InterPro entry have changed between releases. When these changes identify a function, Pfam is notified. To date we have changed the identifiers of 1132 DUF or UPF families indicating that a function has been identified for these families. As of release 33.1, Pfam contains 4244 DUF or UPF families, which is 23% of all Pfam families. This suggests that there are still a lot of uncharacterized families and domains for molecular biologists to study.

We regularly receive feedback from users about families or domains that are missing in Pfam, and typically add many user submitted families at each release. We include the submitters name and ORCID identifier (https://orcid.org/) where available as an author of such Pfam entries. This helps people to get credit for community activities that improve molecular biology databases such as Pfam. One such user submission was from Heli Mönttinen (University of Helsinki) who submitted a large scale clustering of virus families. Based on this clustering we added 88 new families to Pfam. We currently have 144 non-Pfam authors listed by their ORCID and we encourage our users to continue to submit interesting potential new domains and families.

## COVID-19 UPDATES

The SARS-CoV-2 pandemic has mobilized a worldwide research effort to understand the pathogen itself and the mechanism of COVID-19 disease, as well as to identify treatment options. Pfam already provided useful annotation for SARS-CoV-2, but we sought to update our models, family names and annotations for this virus in an effort to help the research community.

**Figure 2.**
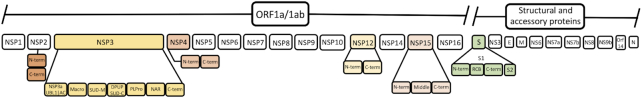
Schematic representation of Pfam coverage of the SARS-CoV-2 proteome. The top row of boxes represent the individual virus proteins processed from the precursor polyproteins. These boxes are coloured when they contain more than one Pfam domain and the individual Pfam entries are expanded below.

We assessed all the protein sequences provided by UniProt via its new COVID-19 portal (https://covid-19.uniprot.org/), identified those which lacked an existing Pfam model, and built new models as required. We now cover almost all gene products encoded by SARS-CoV-2 (Figure 2). Orf10, a small putative protein encoded at the 3′-end of the SARS-CoV-2 genome is the only protein which remains unannotated by Pfam. It was not possible to build a Pfam entry for it since it lacked any detectable homologues in UniProtKB. The mean percentage identity for the full alignments (based on reference proteomes) of Pfam entries that match SARS-CoV-2 proteins is 49% (range 15–96%).

We have standardized our identifier nomenclature and descriptions of the families to ensure they are both correct and consistent. The majority of the family identifiers now begin with either CoV, for coronavirus specific families, or bCoV for the families which are specific to the betacoronavirus clade, which SARS-CoV-2 belongs to. We have also fixed inconsistencies in the naming and descriptions of the various non-structural proteins (NSPs), using NSPx for those proteins encoded by the replicase polyprotein and NSx for those encoded by other ORFs.

### Structural and accessory proteins

One of the most important proteins of this virus is the Spike protein (S), which aids viral entry into the host cell, and is key for its pathogenicity. The models that were already present in the database have been improved and we added a new domain entry. Following this, as the S protein is translated into a large polypeptide which is cleaved by host proteases to produce S1 and S2 peptides, we have now three domains corresponding to S1, the N-terminal domain (Pfam:PF16451), the receptor binding domain (RBD) (Pfam:PF09408) and the new C-terminal domain (Pfam:PF19209). S2 is described in the family Pfam:PF01601, which contains an additional S2′ cleavage site, a fusion peptide (FP), internal fusion peptide (IFP), heptad repeat 1/2 (HR1/2), and the transmembrane domain (TM).

Entities for the other structural proteins in Pfam were updated, such as the Nucleocapsid protein (N) in Pfam:PF00937, the matrix protein (M) Pfam:PF01635 and the envelope E protein in Pfam:PF02723. Additional accessory proteins encoded by coronaviruses, usually called non-structural accessory proteins (NS), although some of them constitute structural parts of the virion, were updated. For example, NS3a corresponds to the betacoronavirus viroporin in the Pfam entry Pfam:PF11289, and Pfam:PF09399 describes the protein 9b encoded within the nucleocapsid gene, which contains a lipid binding domain. The accessory proteins NS7a and NS7b in the entries Pfam:PF08779 and Pfam:PF11395, respectively, are important during the replication cycle. NS8 in Pfam:PF12093 family may modulate viral pathogenicity or replication in favour of human adaptation and it has been to be one of the most important genes in the study of the human adaptation of the virus. The accessory protein NS6 (Pfam:PF12133) is highly conserved amongst SARS-related coronaviruses and it can modulate host immune responses by inhibiting synthesis and signalling of interferon-beta. The family Pfam:PF17635 describes the protein 14 encoded in Orf14 and its function is currently unknown.

### Non-structural proteins

Regarding NSPs from coronaviruses, encoded by ORF1a/1ab (replicase 1a/1ab), we updated the existing entries and added new ones where appropriate. NSPs form the replication–transcription complexes (RTCs) that are essential for the synthesis of viral RNA necessary for the infection process or to avoid the host immune response. Amongst these proteins, NSP3 is the largest replicase product including several conserved domains whose organization differ amongst coronavirus genera (13). It has a crucial function as it cleaves the proteins encoded by the replicase, including NSP3 itself. It is an essential component of the RTC and serves as a scaffold protein to interact with itself and other NSPs. We describe it in separate entries: the existing N-terminal domain Pfam:PF12379 was improved and updated, and includes the NSP3a domain which acts as a scaffold through its interaction with numerous proteins involved in replication and transcription processes and contains the ubiquitin-like 1 (UB1) and glutamic acid-rich acidic (AC) hypervariable domains. NSP3a is essential for localizing the RNA in the replicase–transcriptase complex at the first steps of infection, as it interacts with the nucleocapsid protein N in the nascent replicase/transcriptase complex (14). The other domains encoded in this protein are the Macro domain included in Pfam:PF01661 and SUD-M domain in Pfam:PF11633 that binds single-stranded poly(A). The domain Pfam:PF12124 was updated and is now described as the SUD-C or DPUP domain which binds to single-stranded RNA and recognizes purine bases. The papain-like protease (PLPro) crucial for polypeptide processing is described in Pfam:PF08715; the nucleic acid-binding domain (NAR) belongs to Pfam:PF16251 family and, lastly, the C-terminal domain of NSP3 was added as the new entry Pfam:PF19218. Additionally, we built the N-terminal domain of NSP4 represented in the new family Pfam:PF19217 whilst its C-terminal domain is described in Pfam:PF16348 and the DUF5881 was identified as NSP6 in Pfam:PF19213. The other protease activity in these viruses is described in Pfam:PF05409 usually known as Main protease (M-pro domain) or 3C-like proteinase (3CL-pro), corresponding to NSP5, a member of MEROPS (15) peptidase family C30. Other NSPs were in the Pfam database and most of these families have been extended to include SARS-Cov-2 sequences. For example, Pfam:PF08716 and Pfam:PF08717 corresponds to NSP7 and NSP8, respectively, which both form a hexadecameric supercomplex that adopts a hollow cylinder-like structure that play a role in the stabilization of NSP12 regions involved in RNA binding and are essential for a highly active NSP12 polymerase complex. NSP12 is an RNA-directed RNA polymerase described in two entries, Pfam:PF06478 and Pfam:PF00680 describing the N- and C-terminal domains, respectively (16).

Following annotation updates, NSP9 belongs to the family Pfam:PF08710, which is a single-stranded RNA-binding viral protein involved in RNA synthesis, essential for the coronavirus replication. NSP10 included in Pfam:PF09401 is amongst the more conserved coronavirus proteins. It interacts with NSP14 (Pfam:PF06471) and NSP16 (Pfam:PF06460) and regulates their respective exonuclease (ExoN) and ribose-2′-O-MTase (2′-O-MTase) activities. It has a Tyr-96 specific for SARS-CoV and it is of particular interest as it plays a crucial role in the NSP10–NSP16 interaction and in the activation of the NSP16 2′-O-MTase activity as well as in the NSP10–NSP14 interaction (17).

**Figure 3.**
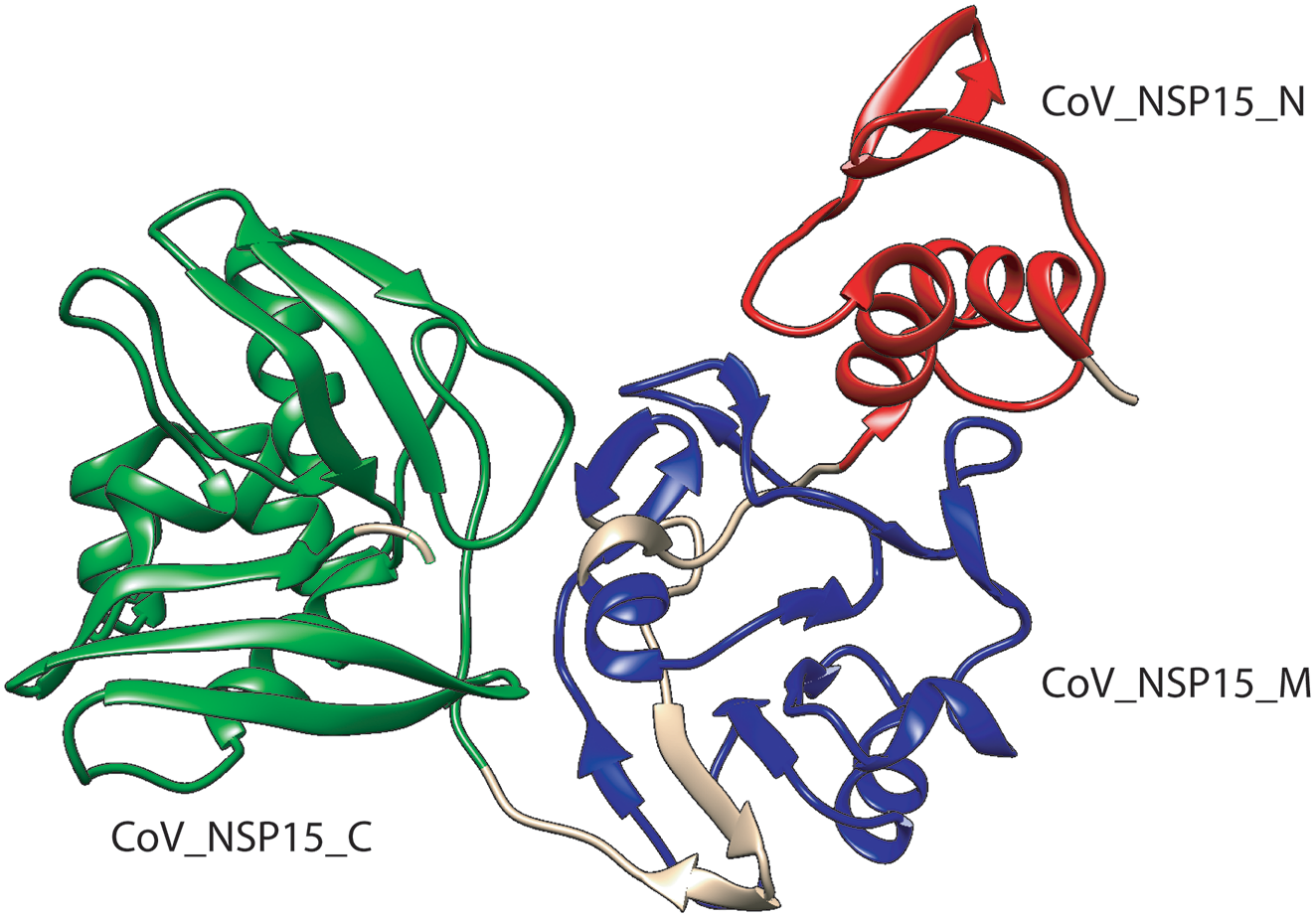
The structure of NSP15 (PDB ID: 6VWW) from Kim *et al.* shows the three new Pfam domains. (i) CoV_NSP15_N (Pfam:PF19219) Coronavirus replicase NSP15, N-terminal oligomerization domain in red, (ii) CoV_NSP15_M (Pfam:PF19216) Coronavirus replicase NSP15, middle domain in blue and (iii) CoV_NSP15_C (Pfam:PF19215) Coronavirus replicase NSP15, uridylate-specific endoribonuclease in green.

Based on recently solved structures of SARS-CoV-2 proteins, we were able to build new families, as is the case of NSP15. We built three new entries representing the three structural domains of the NSP15 protein (Figure 3) based on the structure by Kim *et al.* (18).

## PFAM-B

In addition to our HMM-based Pfam entries (Pfam-A), we used to provide a set of unannotated, computationally generated multiple sequence alignments called Pfam-B. The method used for Pfam-B construction changed significantly over the years. The final prior version of Pfam-B alignments were created from clusters generated by applying the ADDA algorithm (19) to an all-against-all BLAST (20) search of a non-redundant version of UniProtKB, and removing any regions covered by Pfam-A. The version of UniProtKB used in the all-against-all clustering became increasingly out of sync with that used by Pfam. This, combined with the time it took to produce it meant that as of Pfam 28.0 (released in 2015), it was no longer feasible to make Pfam-B (see (3) for a longer discussion on why we stopped producing Pfam-B). We have now devised an alternative method of making Pfam-B that uses the MMSeqs2 software (21) which has a much lower computational cost. The new pipeline was also designed to produce alignments that are more likely to represent new domain families.

Pfam-B was created by clustering pfamseq sequence segments longer than 50 residues not covered by Pfam-A domains, using MMSeqs2 run with the cluster option and bidirectional coverage mode. Multiple sequence alignments were generated for each cluster with more than 20 sequences using FAMSA (22). This resulted in 136,730 Pfam-B families that on average contain 99 sequences (maximum 40 912) and are 310 positions wide (maximum 29 216).

The Pfam-B alignments are presently only released as a tar archive on the Pfam FTP site (file Pfam-B.tgz). We have not built profile HMMs for the entries or integrated them into the Pfam website. The entries are sorted such that the first entries have an optimal combination of size and conservation and would therefore have the highest chance of representing novel and useful domain families.

## PFAM TYPE DEFINITIONS

Pfam type definitions divide entries into one of six types and they can help users in selecting which Pfam families to use in their analyses. Over the past year we have made numerous changes and updates to the Pfam type definitions for families (Table 1). In particular, we have carried out a large scale screen of Pfam families using the ncoils software (23) to identify families with a high proportion of predicted coiled-coil, and after inspection of such families, we were able to change their type.

**Table 1.** The number of each Pfam type for the last two Pfam releases 32.0 and 33.1

Type	Pfam release 32.0	Pfam release 33.1
Family	11 177	11 242
Domain	6248	6406
Repeat	260	268
Coiled-coil	95	171
Motif	82	91
Disordered	67	81

We were interested to see if we can improve the definitions of the other types in Pfam. We have investigated the Disorder and Repeat types within Pfam and report these results below.

## DISORDER IN PFAM

**Figure 4.**
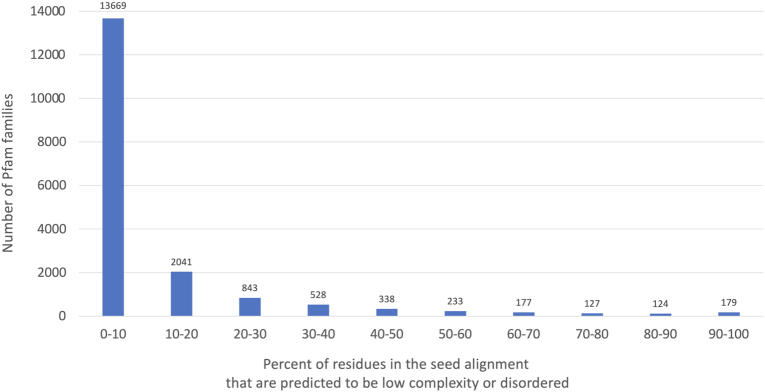
Percent of residues in the seed alignment of Pfam entries that are low complexity or disordered as predicted by segmasker and MobiDB-lite, respectively.

We investigated the level of predicted disorder across all Pfam families. The mean percentage identity for full alignments (based on reference proteomes) of Pfam entries with type Disordered is 55% (range 18–94%). For each family we determined the fraction of residues in the seed alignment that were predicted to be low complexity by segmasker (24) or disordered by MobiDB-lite (25). A histogram of scores is shown in Figure 4. There are 303 Pfam families that have over 80% of seed alignment residues predicted as disordered/low complexity, and yet only 81 are currently classified as disordered. We have begun to reclassify these families for release 34.0.

## REPEATS IN PFAM

To investigate the Repeat type entries in Pfam, we compared them to the gold-standard repeat database RepeatsDB (26). This resource contains a rigorous structural classification of repeat proteins from the PDB and places each into a repeat ontology. Repeat regions in RepeatsDB are detected by structure and annotated with the position of each repeated unit. The comparison to Pfam highlights the differences between sequence- and structure-based repeat identification, domain annotation as well as classification, and is relevant in the context of our ongoing effort of improving repeat definitions.

**Figure 5.**
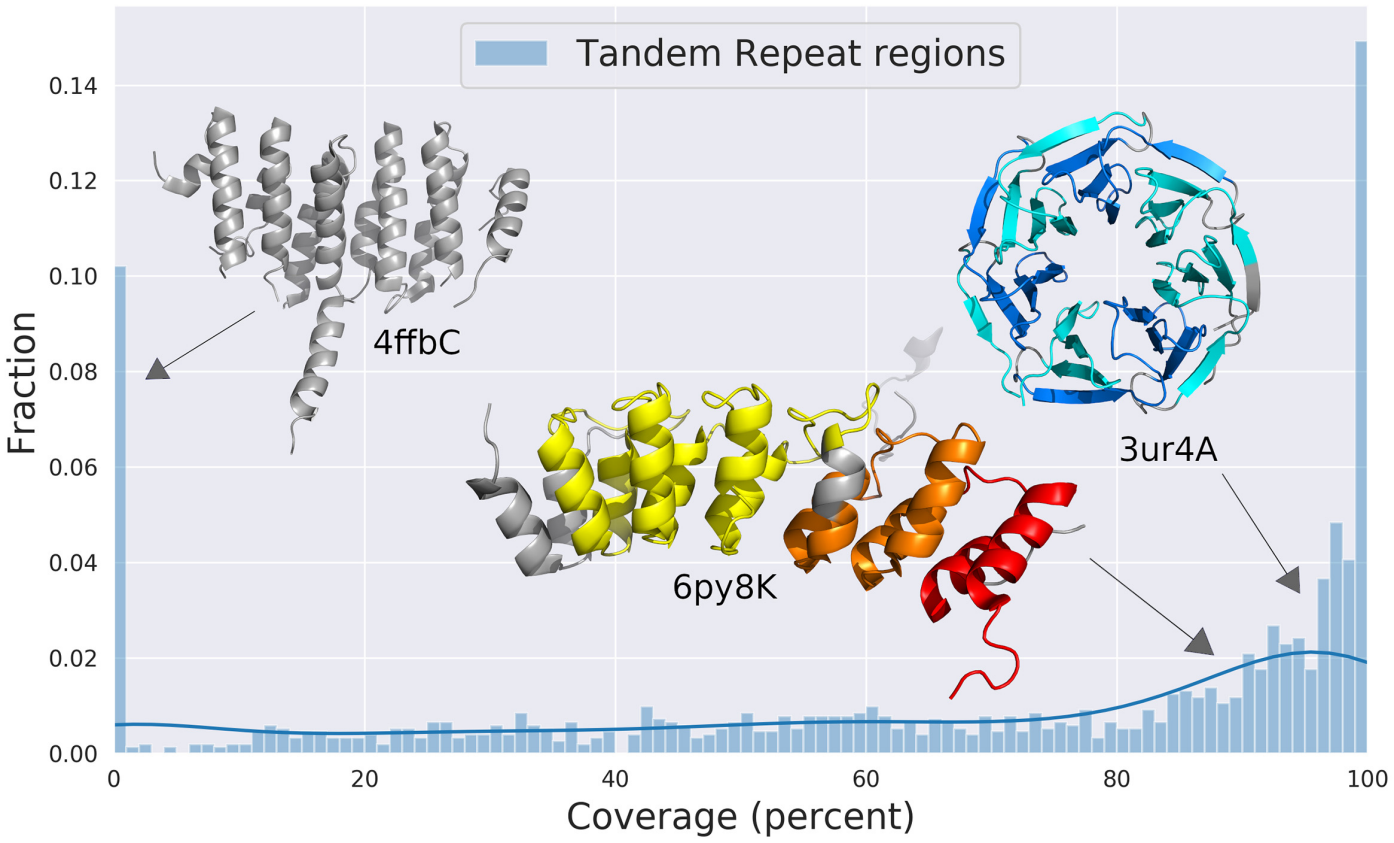
Pfam coverage of repeat regions in UniProtKB entries from RepeatsDB. Three examples are shown represented by their PDB structures. On the top left PDB ID: 4ffb, chain C, mapping to a region of the HEAT repeats in UniProtKB:P46675 (residues 1–272), with Pfam coverage 0%. In the centre, the Ankyrin region of UniProtKB:P46531, PDB ID: 6py8, chain K (residues 1759–2127), with Pfam coverage 89.4%. Three Pfam domains are detected: Pfam:PF12796 in yellow, Pfam:PF13637 in orange and Pfam:PF00023 in red. On the top right, PDB ID: 3ur4, chain A, mapping to the β-propeller UniProtKB:P61964 (residues 24–334), with Pfam coverage 93%. Only one type of Pfam domain is detected (Pfam:PF00400), shown in alternating shades of blue to facilitate the visualization of the Pfam model phase.

Pfam covers 64.2% of the repeat regions found in RepeatsDB (Figure 5) and shows a bimodal distribution, with peaks at 0 and 100%. Therefore, most RepeatsDB entries (1415 UniProtKB proteins) are either fully characterized or uncharacterized by Pfam families. This analysis allowed us to identify candidates for future Pfam curation, that present 0% coverage, such as the STU2 protein (UniProtKB:P46675) containing HEAT repeats (Figure 5), the β-propeller domain in DDB1- and CUL4-associated factor 1 (UniProtKB:Q9Y4B6) and the LRR domain in Ran GTPase-activating protein 1 (UniProtKB:P41391). These sequences will be aligned with structurally similar entries in RepeatsDB to derive the input alignments for Pfam, and the eventual overlap with existing sequence models (e.g. the existing Pfam LRR and HEAT repeat domains) will be investigated and resolved.

We investigated the number and types of Pfam domains detected in RepeatsDB regions. A total of 573 Pfam domains in 106 Pfam clans are mapped to repeats, of which only 91 (15.9%) are of type Repeat. A total of 176 (30.7%) are of type Family, 303 (52.9%) Domain, 1 Coiled-coil (mapping to entries in RepeatsDB fibrous structures) and 2 Motif. The large majority of Family and Domain types may be justified by the fact that RepeatsDB includes a class, Beads-on-a-string, whose repeat units are large enough to fold independently (26), likely corresponding to Pfam type domain rather than type repeat. Indeed, class V entries mostly (86.5%) map to Domains in Pfam. However, RepeatsDB elongated structures, i.e. the most canonical repeats, including solenoids (26), are still mostly mapped to non-Repeat Pfam types (22.2% are Repeat), including the two Motif entries.

These data will support the revision of Pfam types assignment in repeat entries, also in light of the RepeatsDB units to Pfam domains correspondence. Pfam models may be defined in different ways: (i) they may map to the entire repeat region, such as the Ricin-type beta-trefoil lectin domain (Pfam:PF00652) in the rRNA N-glycosidase (UniProtKB:B7 × 8M2). The model for this domain was initially designed to correspond to single units, but it was later revised and updated to the current version to increase its sensitivity, supported by the observation that this type of repeat region usually includes a tandem repeat of three units. In this case, the type assignment to Domain should be kept. (ii) Pfam models may map to each repeat unit, with the same phase, as the RCC1 repeats (Pfam:PF00415) in the human Regulator of chromosome condensation (UniProtKB:P18754), or with a different phase, as the WD domain (Pfam:PF00400) in the WD repeat-containing protein 5 (UniProtKB:P61964). In the latter, whilst the RepeatsDB phase is defined as corresponding to a single entire blade of the β-propeller, the Pfam model maps to a segment including most of each blade and the first β-sheet of the following one (Figure 5). This type of unit is called ‘Velcro’ and has stabilizing functions (27). The two phases are therefore related to two different concepts, and these types of examples highlight cases where the structural repeat patterns may be used to revise the sequence one or *vice versa*. (iii) Finally, different Pfam models may map to the same repeat region, and correspond to different number of units, such as in the Ankyrin region (Figure 5) of the Neurogenic locus notch homologue protein 1 (UniProtKB:P46531), where the three types of Pfam models detected (Pfam:PF00023, Pfam:PF13637 and Pfam:PF12796) map respectively to one, two and three repeat units. With this arrangement, one unit and a fragment are actually skipped by Pfam models; these cases will require an extensive investigation of the optimal number of units included in Pfam models and the potential overlaps.

## DISCUSSION

The Pfam sequence and residue coverage of UniProtKB has remained fairly constant over the past few years at ∼77 and ∼53%, respectively. This means that although Pfam aims to be comprehensive, there remains a significant area of sequence space that has no annotation by Pfam. As UniProtKB grows, it becomes progressively harder to increase the coverage as the larger ubiquitous families have already been built, and newer families tend to have a smaller taxonomic range. Nevertheless there are still many important families left to build, and we plan to concentrate our efforts on family building for the next release. New families will be built from a range of sources such that we represent the diversity in the tree of life. Sources will include Pfam-B clusters, which we re-introduced into Pfam in the current release (version 33.1). We will also continue to build families from metagenomic sequence clusters, PDB structures and community submissions.

In response to the COVID-19 pandemic, we have revised all existing families that match SARS-CoV-2 proteins, and built new profile HMMs to cover regions previously unannotated by Pfam. All proteins in the SARS-CoV-2 proteome are covered by Pfam apart from the putative protein encoded by Orf10, which lacked any detectable homologues in UniProtKB. We hope our models help the research community to identify and annotate coronavirus sequences. Additionally, multiple sequence alignments generated using Pfam HMMs may prove useful in tracking the evolution of coronaviruses.

Since the last release, we have updated some of our type definitions, particularly those that are predicted to have coiled-coil regions. We have also identified a set of families that have a high proportion of disordered residues, and plan to reclassify these families as type disorder for the next Pfam release. We have also compared Pfam to the RepeatsDB database. Using the comparison, we will look to change the type of some of the Pfam entries to repeat, incorporate additional RepeatsDB regions into Pfam and revise the domain boundaries on some of our repeat entries.
